# Immunosuppression and Surgery‐Free Interval in Granulomatosis With Polyangiitis Airway Stenosis

**DOI:** 10.1002/lary.70249

**Published:** 2025-11-03

**Authors:** Sydney J. Torres, Andrew J. Neevel, Julia A. Ford, Lawrence Kashat, Norman D. Hogikyan, Robbi A. Kupfer, Robert J. Morrison

**Affiliations:** ^1^ University of Michigan Medical School Ann Arbor Michigan USA; ^2^ University of Michigan Department of Otolaryngology‐Head and Neck Surgery Ann Arbor Michigan USA; ^3^ Division of Rheumatology University of Michigan Department of Internal Medicine Ann Arbor Michigan USA; ^4^ MidMichigan Ear, Nose, and Throat East Lansing East Lansing Michigan USA

**Keywords:** granulomatosis with polyangiitis, immunotherapy, laryngotracheal stenosis, recurrence, rituximab, subglottic stenosis, tracheal stenosis

## Abstract

**Objectives:**

Subglottic and tracheal stenosis (SGS, TS) are severe manifestations of granulomatosis with polyangiitis (GPA), often‐requiring endoscopic airway intervention and systemic immunosuppression. Rituximab (RTX) has shown efficacy for systemic GPA, but its role in SGS and TS remains unclear, with reports of both benefit and relapse. This study evaluated the impact of RTX and cyclophosphamide (CTX) on surgery‐free intervals (SFIs) in GPA‐associated SGS and TS.

**Methods:**

A retrospective chart review was conducted on GPA patients with SGS or TS treated at a tertiary center between 1992 and 2023. Therapeutic RTX exposure was defined as 3–9 months post‐induction or within 1 year of maintenance, and CTX during regular dosing before transitioning to another maintenance regimen. SFIs, calculated as time between endoscopic interventions, were compared with and without RTX and CTX exposure using weighted *t*‐tests.

**Results:**

A total of 55 patients met inclusion; 44 (80%) underwent at least one endoscopic intervention and 34 of those patients (77%) received RTX and/or CTX. Median follow‐up time was 10.2 years (range 0.8–29.5 years). Mean SFIs were significantly longer during therapeutic RTX exposure or remission (45 ± 8 months) versus non‐therapeutic intervals (20 ± 6 months) (*p* = 0.046). Prior RTX or CTX use, though not definitionally therapeutic, significantly increased mean SFI from 22 to 25 months compared to no exposure (*p* = 0.029). CTX alone did not significantly increase SFI.

**Conclusion:**

Rituximab may contribute to longer intervals between endoscopic airway intervention and delay relapse in GPA‐related airway stenosis, supporting its role in stabilization of airway manifestations. Multidisciplinary management remains essential for this life‐threatening GPA manifestation.

**Level of Evidence:**

3.

## Graphical abstract

Subglottic and tracheal stenosis are severe manifestations of granulomatosis with polyangiitis (GPA) that often require both endoscopic intervention and systemic immunosuppression. Rituximab may help stabilize GPA‐related airway disease by prolonging the interval between endoscopic procedures and delaying relapse.

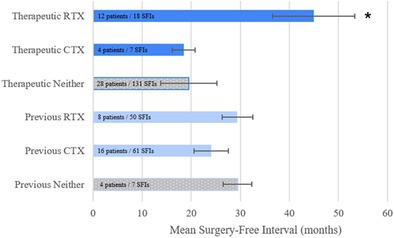

## 1 Introduction

Proximal airway stenosis is a potentially life‐threatening and debilitating condition with autoimmune, iatrogenic, and idiopathic etiologies. The disease process is often categorized by the specific subsite involved, including the supraglottic, glottic and/or subglottic regions of the larynx (laryngeal stenosis), the trachea proper (tracheal stenosis), or it may be referred to more globally as laryngotracheal stenosis (LTS). LTS is present in 10% of patients with granulomatosis with polyangiitis (GPA; previously Wegener's granulomatosis), an antineutrophil cytoplasmic antibody (ANCA)‐associated vasculitis (AAV) [1]. GPA can be characterized as limited (isolated to the upper airway, eyes, and ear, nose, and throat manifestations, including LTS) and generalized (typically renal, pulmonary, or other systemic involvement) [2, 3, 4, 5]. Manifestations of GPA have also been characterized as both vasculitic (e.g., renal, alveolar, mononeuritis multiplex, scleritis, and constitutional involvement) and granulomatous (e.g., orbital masses, pulmonary nodules, pachymeningitis, and granulomatous involvement of the upper aerodigestive tract, including chronic sinusitis, nasal crusting, septal perforation, and LTS) [6, 7, 8]. The limited and granulomatous manifestations, including LTS, are thought to be more refractory to treatment than the generalized and vasculitic manifestations, complicating management strategies [2, 9, 10, 11, 12, 13, 14]. Active airway inflammatory disease can be challenging to distinguish from subsequent scarring, which adds to clinical complexity [2, 15, 16, 17, 18].

GPA‐associated laryngotracheal stenosis (GPA‐LTS) is characterized by a relapsing and remitting course. Endoscopic airway interventions are the standard approach for managing GPA‐LTS, which may include laser or cold knife excision, balloon dilation, and anti‐fibrotic adjuvants such as steroid injection and mitomycin‐C application [19]. Endoscopic management often requires repeated excision and dilation with frequency dependent upon stenosis recurrence and the patient's clinical symptoms. Surgery–free interval (SFI), the time between endoscopic interventions, is a commonly used proxy for LTS clinical disease aggressiveness [20, 21, 22, 23]. For refractory cases of LTS, open cricotracheal resection may be considered [24, 25]. However, in active inflammatory flares of GPA‐LTS, definitive surgical intervention is typically contraindicated due to the risk of reconstructive failure, and even less invasive endoscopic procedures are avoided if possible during active flares due to perceived negative impact upon effecting surgical change [16, 19, 26, 27].

While systemic therapies such as cyclophosphamide (CTX) and rituximab (RTX) have demonstrated efficacy in managing non‐airway manifestations, their long‐term impact on GPA‐LTS remains uncertain [10, 12, 13, 28, 29, 30, 31, 32, 33, 34, 35]. The airway‐specific effect of systemic immunosuppression and potential to slow or prevent recurrence of stenosis remains unclear due to disease and management heterogeneity and limited sample sizes in recent retrospective studies [4, 9, 19, 36, 37, 38].

No clear guidelines exist for the treatment of GPA‐LTS [19]. Multiple studies, including the RAVE trial, suggest RTX is beneficial for treating granulomatous and relapsing forms of GPA; however, conflicting evidence exists regarding its efficacy in airway stenosis specifically [10, 12, 14, 33, 34]. Emerging evidence highlights hesitancy in treating GPA‐LTS with RTX due to varying results and reliability in LTS response. Observations that airway stenosis can develop or progress despite treatment with RTX or independently of other systemic disease manifestations have led to speculation that its pathophysiology may involve unique processes beyond typical granulomatous inflammation [5, 10, 17, 30, 39, 40, 41, 42, 43]. However, other studies suggest favorable remission induction and maintenance of GPA‐LTS with RTX treatment, often even with refractory forms of disease [2, 9, 14, 31, 44]. Additionally, there is limited applicability of prior studies due to high heterogeneity from small sample sizes, inclusion of other immunological diseases, focus on procedural interventions rather than systemic immunotherapy in GPA‐LTS, and inconsistent treatment outcomes.

It remains unclear if the progression of GPA‐LTS can be modified by systemic treatment and if the aggressiveness of the airway manifestations should be an individually considered factor in the initiation or maintenance of systemic treatment for patients with GPA. An improved understanding of GPA‐LTS recurrence at multiple stages of disease—presentation, induction, and remission—would inform multidisciplinary and multimodal management strategies. Our study aims to address these gaps by characterizing the impact of systemic rituximab and cyclophosphamide exposure on the surgery‐free interval in GPA‐associated airway stenosis.

## 2 Materials and Methods

### 2.1 Patient Population

This study was determined to be exempt by the Institutional Review Board at the University of Michigan (HUM00188222). DataDirect, an institutional medical health data tool, was used to retrieve all patients treated at the University of Michigan from December 1992 to February 2023 diagnosed with both laryngotracheal stenosis and granulomatosis with polyangiitis. These patients were identified using International Classification of Diseases, 9th edition (ICD‐9) and International Classification of Diseases, 10th edition (ICD‐10) codes for laryngeal stenosis (J95.5, J95.0, J38.6, J39.8, 478.74, 997.32, 519.19, 519.1, and 519.02) and granulomatosis with polyangiitis (M31.3, I77.82). A preliminary list of 204 patients was retrieved and manual chart review was performed to screen patients for inclusion. Patients were included if they had either a confirmed diagnosis or a strong probability of granulomatosis with polyangiitis by a rheumatologist. Strong probability was defined as patients who were ANCA‐negative but had multi‐system inflammation and underwent treatment. Laryngotracheal stenosis was diagnosed by a fellowship‐trained laryngologist via flexible laryngoscopy, laryngeal videostroboscopy, or bronchoscopy. Patients with presumptive idiopathic disease (ANCA‐negativity with laryngotracheal stenosis in isolation) or patients who had incomplete records to determine other manifestations of systemic disease, treatment course, or outcomes were excluded.

### 2.2 Exposures and Outcomes

Demographic data were collected, including age, sex, relevant comorbidities, and ANCA status. Airway‐related otolaryngologic procedures and dates were collected, including operating room (OR) endoscopic stenosis excision and dilation, tracheostomy, and in‐office steroid injection. Surgery‐free interval (SFI) was defined as the time between OR endoscopic interventions. Patients required at least 2 endoscopic interventions to calculate SFI. If exact months and dates were unavailable, January and the first day of the month were assumed for calculations, respectively. Medication and surgical treatments completed at outside institutions prior to, or while being treated at Michigan Medicine, were included if records were available.

Rituximab (RTX), cyclophosphamide (CTX), and oral corticosteroid administration dates, course completion dates, dosage, rationale for starting and stopping, and side effects were collected via chart review. Therapeutic exposure to RTX and CTX was defined conservatively based on expected duration of therapeutic effectiveness. SFIs within 3–9 months post‐induction of rituximab, within 1 year from maintenance doses, or if clinically considered to be in remission by a rheumatologist after disease modification were considered actively therapeutic [45, 46].

Exposure to rituximab required induction dosing with either 1000 mg/dose for 2 doses given 2 weeks apart or 375 mg/m^2^ weekly for 4 weeks (RAVE trial protocol dosing). Rituximab and/or cyclophosphamide is used for induction therapy of severe disease alongside glucocorticoids, though given its more favorable side effect profile, rituximab has tended to be the induction agent of choice by rheumatology at our institution since publication of the RAVE trial in 2010. Continuation of regular rituximab, cyclophosphamide, or steroid dosing was individualized and variable based on clinical response and ongoing disease activity.

Patients were considered therapeutic while on maintenance rituximab if they received additional infusions of 500 mg or 1000 mg every 6–12 months. SFIs were considered actively therapeutic during active cyclophosphamide dosing, defined as immediately following induction and prior to transition to an alternate maintenance regimen. SFIs were considered positive for steroid treatment if taking oral corticosteroids > 50% of the SFI or receiving > 3 SILSI during their SFI. Many of the intervals designated as “non‐therapeutic” include dosing with other medications that included steroids, methotrexate, and azathioprine.

### 2.3 Data Analysis

Independent *t*‐tests were used to compare mean SFI in various treatment conditions. These included SFIs actively therapeutic on RTX, CTX, and steroids as well as previously treated with RTX and CTX. Mean SFIs were weighted based on the number of SFIs available. A paired t‐test was performed for patients with both pre‐treatment and post‐treatment calculable SFIs. A pre‐determined *P*‐value of < 0.05 indicated significance for all statistical analyses, which were 1‐sided. Levene's test for variance determined whether parametric or non‐parametric tests were used. Robust standard errors were calculated to adjust for individual patient clustering in those with multiple SFIs. Multiple linear regressions was performed to screen for a confounding effect of steroid exposure. Data analysis was performed in SPSS Statistics 29.0.0.0 (IBM). Additional case‐by‐case analysis was performed.

## 3 Results

**FIGURE 1:**
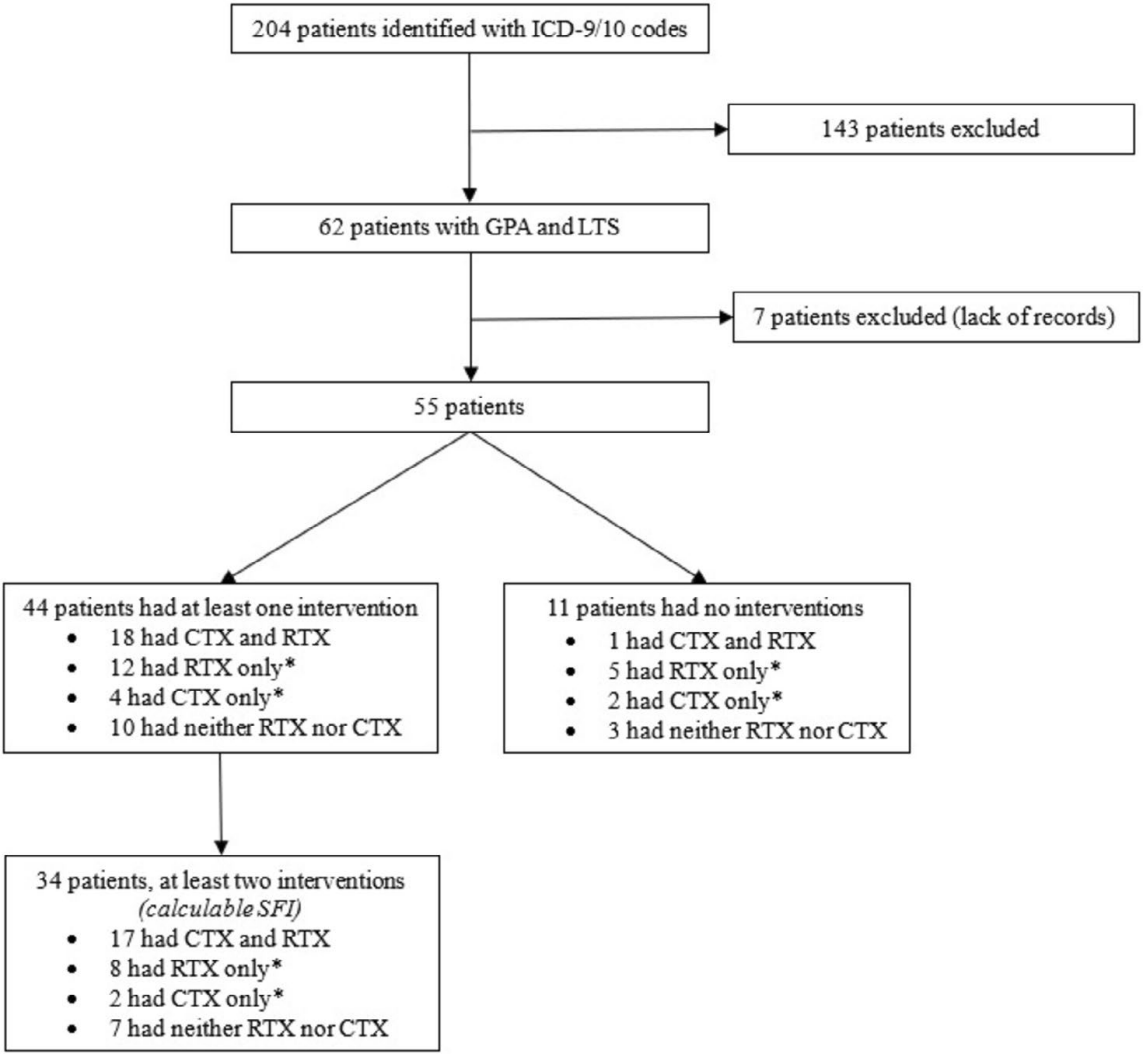
Inclusion and exclusion with number of patients receiving immunosuppression. International classification of diseases 9th/10th editions. GPA = granulomatosis with polyangiitis. LTS = laryngotracheal stenosis. SFI = surgery free interval. Intervention = endoscopic excision and dilation. *“only” does not exclude use of other immunosuppressive agents.

A total of 55 patients with laryngotracheal stenosis and a diagnosis of GPA were included (Figure 1). Patients were predominantly female (76%), and the majority were ANCA positive (78%) (Table 1). A total of 44 (80%) patients underwent at least one endoscopic intervention, and 34 of these patients (77%) were treated with RTX and/or CTX during their follow‐up time (Figure 1, Table 2). A total of 34 patients (62%) had at least two endoscopic interventions and therefore had a calculable SFI. A total of 12 patients (36%) were therapeutic on RTX or CTX or in remission during a calculable SFI. Median follow‐up time from first endoscopic intervention to most recent otolaryngology or rheumatology follow‐up was 10.2 years (range 0.8–29.5 years).

**TABLE 1:** Patient demographics and disease characteristics.

	All patients (*n* = 55)
Age (years)	53 (23–83)
Sex	
Female	42 (76%)
Male	13 (24%)
ANCA Status	
Negative	12 (22%)
Positive	43 (78%)
cANCA Positive Only	21 (38%)
pANCA Positive Only	17 (31%)
Both pANCA and cANCA Positive	2 (4%)
PR3 Confirmed if cANCA Positive	18 (78%)
MPO Confirmed if pANCA Positive	16 (84%)
ANCA Unknown	3 (5%)
Concurrent Disease Involvement	
Renal	13 (24%)
Pulmonary	25 (45%)
Ear	23 (42%)
Eye	12 (22%)
Sinonasal	38 (69%)
Joint	21 (38%)
Airway only	6 (11%)
Endoscopic Interventions	
0	11 (20%)
1	10 (18%)
2 to 5	22 (40%)
6 to 10	6 (10%)
11+	6 (11%)
Mean Follow‐Up Duration (years) (*first intervention to most recent visit*)	10.2 (0.8–29.5)

*Note*: Data presented as median (range) or *n* (%).

Abbreviations: c/pANCA = cytoplasmic/perinuclear antineutrophil cytoplasmic antibody; MPO = myeloperoxidase; PR3 = anti‐proteinase 3.

**TABLE 2:** Number of patients who received rituximab and/or cyclophosphamide by number of airway interventions.

	Both RTX and CTX	RTX only*	CTX only*	Neither RTX nor CTX	Total
No intervention	1	5	2	3	**11**
One intervention only	1	4	2	3	**10**
Two + interventions only (*calculable SFI*)	17	8	2	7	**34**

Abbreviations: CTX = cyclophosphamide; RTX = rituximab; SFI = surgery free interval.

* Only does not exclude other immunosuppressive agents.

**FIGURE 2:**
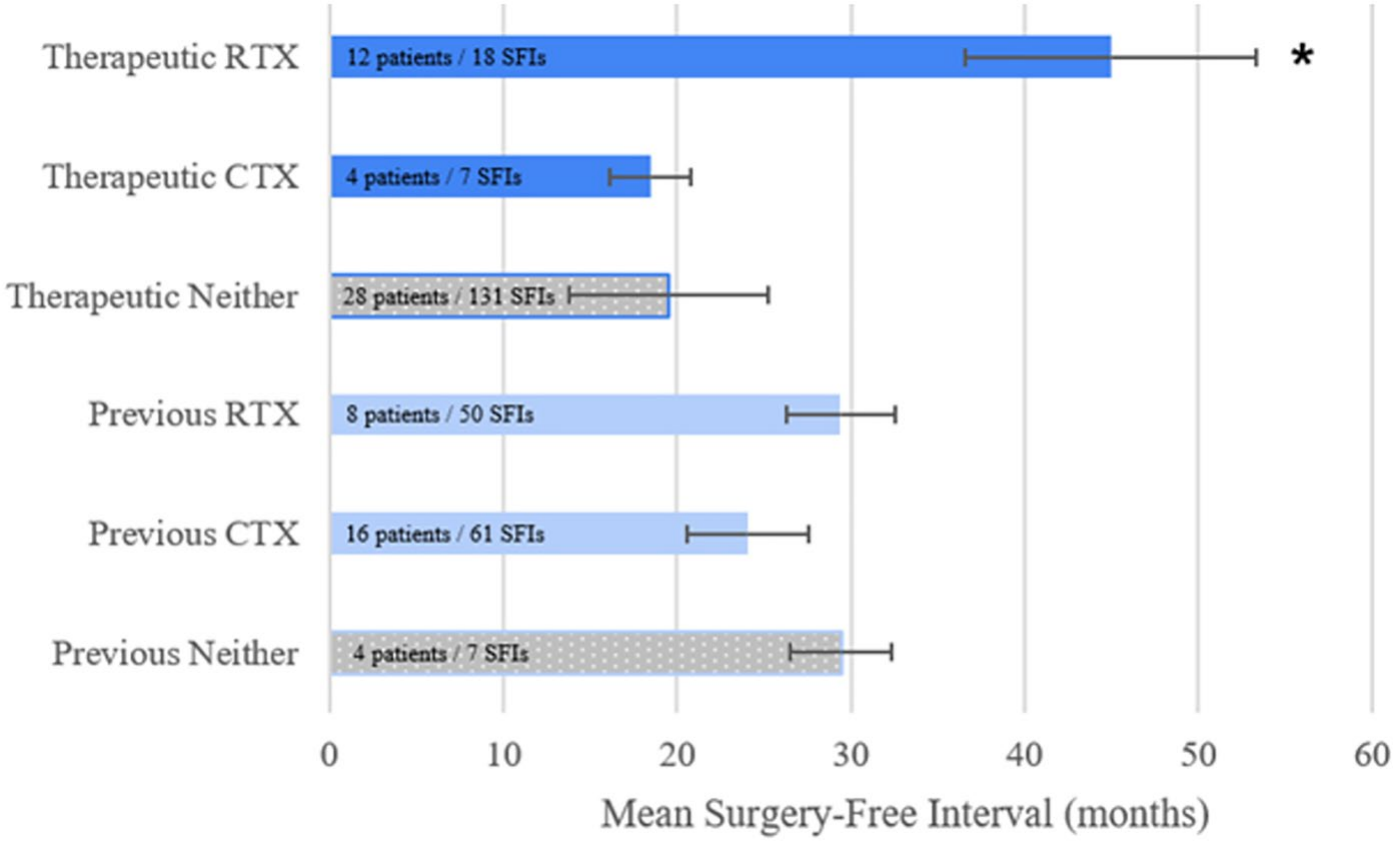
Surgery free‐interval in patients treated with rituximab and cyclophosphamide therapeutic = within 3–9 months post‐induction of rituximab, within 1 year from maintenance rituximab doses, currently being treated with cyclophosphamide, or clinically considered to be in‐remission. Previous = previously treated with rituximab or cyclophosphamide, Neither = no rituximab or cyclophosphamide, but does include other immunosuppressive agents. RTX = rituximab, CTX = cyclophosphamide. * = *p* < 0.05 for 1‐sided t‐test compared to “Neither”. Error bars: Robust standard error. [Color figure can be viewed in the online issue, which is available at www.laryngoscope.com]

**FIGURE 3:**
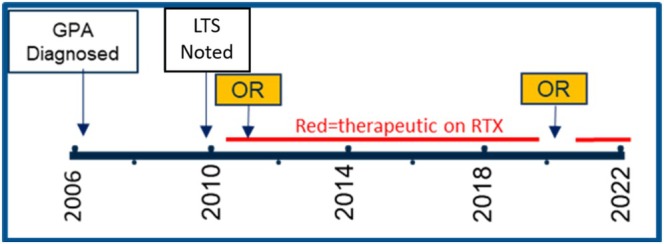
Patient with GPA‐LTS recurrence after discontinuation of rituximab during the COVID‐19 pandemic. GPA = granulomatosis with polyangiitis, LTS = laryngotracheal stenosis, OR = endoscopic operating room intervention, RTX = rituximab. [Color figure can be viewed in the online issue, which is available at www.laryngoscope.com]

Mean SFI of all equally weighted intervals was greater when therapeutic on RTX or during disease remission post‐treatment with RTX (45 ± 8 months, mean ± robust standard error) compared to non‐therapeutic intervals (20 ± 6 months) (*p* = 0.046, Figure 2). Disease remission post‐treatment was included in this group if remission was clinically attributed to RTX treatment by their treating rheumatologist. Three of the patients with calculable SFIs achieved remission and long‐term control of LTS from RTX and were able to discontinue annual dosing without recurrence. One patient maintained long‐term control of subglottic stenosis on repeated therapeutic dosing of RTX for 10 years (Figure 3). When RTX infusion was delayed during the COVID‐19 pandemic, the patient required repeat endoscopic laser excision and dilation within 1 year. A subset of five patients had measurable SFIs before treatment and while therapeutic on either RTX or CTX, one patient receiving both treatments. Paired analysis revealed a significant, though small, increase in mean SFI from 22 to 25 months post‐RTX treatment (*p* = 0.029).

Mean SFI when therapeutic on CTX was not significantly greater (18 ± 2 months) than no treatment (*p* = 0.457). There was no difference in mean SFI between patients previously treated with RTX (29 ± 3 months) or CTX (24 ± 4 months) compared to those never treated (29 ± 3 months). This comparison excluded the three patients who achieved long‐term control of LTS and discontinued RTX. There was a significantly shorter SFI in patients with prior RTX or CTX treatment when no longer definitionally therapeutic, versus those never treated with RTX and CTX (*p* = 0.045) (Figure 2).

Nine patients underwent SILSI, while only 3 patients had calculable SFIs while undergoing > 3 injections. A total of 41% of SFIs had concurrent steroid exposure, but there was no significant improvement in SFI (*p* = 0.422). When correcting for steroid and cyclophosphamide exposure on multivariate linear regression, rituximab exposure was independently associated with improvement in SFI (*p* = 0.001).

## 4 Discussion

In this retrospective study, we evaluated the impact of rituximab (RTX) and cyclophosphamide (CTX) on the surgery‐free interval (SFI) in patients with granulomatosis with polyangiitis‐associated laryngotracheal stenosis (GPA‐LTS). To the authors' knowledge, this is the largest cohort of patients with this uncommon manifestation of a rare autoimmune disease to assess the effect of systemic treatment on airway disease. Our findings suggest that systemic immunosuppression with RTX is associated with prolongation of SFI compared to periods without therapeutic systemic therapy. Specifically, patients on RTX or in remission had a mean SFI more than twice as long (45 months) as observed during non‐therapeutic periods (20 months). Therapeutic definitions were chosen conservatively, which may have included treatment effects during non‐therapeutic periods and decreased the observed difference. Furthermore, because patients who achieved disease stability on RTX often did not require further intervention by their last follow‐up, the SFIs reported may underestimate the extended intervals that could be captured if follow‐up continued beyond the study period. Association with rituximab exposure and longer SFI was maintained when adjusting for steroid use.

Paired analysis of a subset of patients with both pre‐RTX and therapeutic RTX SFIs showed a small, but statistically significant increase from 22 to 25 months. Limited follow‐up in these patients could also lead to underestimating the RTX effect on SFI. This analysis suggests that systemic disease treatment may play a crucial role in stabilizing LTS and reducing the frequency of endoscopic airway interventions.

These findings align with the existing literature highlighting the complex interplay between systemic disease activity and localized airway involvement in GPA. While proximal airway stenosis has historically been viewed as localized, treatment‐resistant manifestations of GPA, our results suggest that systemic immunosuppression with RTX is associated with slower stenosis progression [2, 4, 9, 10, 42, 47]. Multiple studies suggest benefit from combined medical and endoscopic airway interventions, but few studies have previously assessed time between surgical interventions, which serves as a proxy for symptomatic recurrence [2, 16, 19]. Previous studies have reported mixed outcomes regarding the role of systemic therapy in LTS. Our study adds to the growing evidence that RTX, a B‐cell depleting agent with proven efficacy in GPA, may be beneficial in prolonging symptom‐free periods and reducing the need for frequent endoscopic interventions in patients with GPA‐associated LTS. While RTX is already the first‐line treatment for systemic GPA, our study suggests that patients with limited laryngotracheal disease may also benefit. Notably, some patients in our cohort achieved long‐term proximal airway disease control with RTX, including one patient with sustained remission for over a decade. We included patients who achieved systemic disease remission in the therapeutic RTX group to reflect the disease‐modifying effect of rituximab. However, other patients who were treated with RTX and CTX and discontinued without definitive clinical remission did not experience a longer mean SFI. For those patients treated with CTX initially, there was no difference between SFI when therapeutic on CTX. The shorter mean SFI observed in patients previously treated with RTX or CTX, though not therapeutic during later SFIs, may reflect a subset with more active or refractory disease reliant on immunosuppressive therapy.

Despite these promising findings, several limitations warrant consideration. The retrospective design inherently introduces selection bias and limits the ability to establish causality. These patients undergo complex individualized decision‐making regarding disease management with their surgeon and evaluating rheumatologist that may introduce bias. Additionally, the relatively small sample size, several patients lost to follow‐up, and the single‐center nature of our study may restrict the generalizability of our findings. While the number of calculable SFIs was limited based on stringent definitions for therapeutic immunosuppressive dosing, the total number of patients was on par if not larger than most existing studies. While rigid therapeutic definitions ensured consistency, they limited analysis and the ability to perform subgroup comparisons. While the assumed RTX therapeutic effect is within the window of 3–9 months, B‐cell depletion can occur as quickly as 3 days, showing varying response rates to medications [45, 46, 48]. This variable time to depletion may factor into varied responses to RTX, including reported progression despite RTX treatment, such as the case report by Asim Khan and other studies suggesting LTS as an independent predictor of GPA relapse after RTX treatment [5, 10, 14, 17, 30, 39, 40, 41, 42, 43]. Variability in surgical techniques, previous or concurrent adjunctive immunosuppressant medications, disease heterogeneity, and provider preferences may also have an influence [2, 16, 37, 49, 50]. Decisions to pursue systemic treatments in patients are very individualized with heterogeneous responses to medications. Many of these patients were also treated with methotrexate or azathioprine, especially during non‐therapeutic RTX windows, but these data were too variable for meaningful correction. We corrected for steroid exposure, but this was a simplified categorical analysis that did not incorporate the variations in steroid dose and intermittent bursts. Only three patients had a calculable SFI with SILSI exposure, though 6 more had recent SILSI treatments and no return to OR. Additional follow‐up could elucidate the effect of SILSI in this population. A further limitation is the lack of available data and standardized assessment of active airway inflammation versus scarring and percentage of airway stenosis or patency at the time of intervention, which could provide greater insight into the mechanisms underlying disease progression and response to therapy. SFI has an inherently subjective element, as the decision for surgery is typically a shared one between surgeon and patient. It is based on symptom burden from airway stenosis, physiologic measures, and the perceived safety of the airway as assessed by anatomic evaluation by endoscopy or radiographic studies. These factors can vary between patients, influencing both the follow‐up timeline and threshold for intervention [51]. Furthermore, the heterogeneous use of RTX and CTX, often dictated by broader systemic disease activity rather than LTS alone, complicates the interpretation of treatment effects on airway disease.

Moving forward, prospective studies with standardized treatment protocols and objective measures of airway inflammation and obstruction would assist in further delineating the role of systemic therapy in GPA‐LTS. The integration of patient characteristics and subgroup analysis including biomarkers, age at disease onset, SFIs during pre‐immunosuppression, induction, and maintenance therapy, and concomitant immunosuppressive use or serial intralesional steroid injections (SILSI) may help refine treatment strategies and identify patients who would most benefit from systemic immunosuppression. This may help elucidate patient characteristics for those who achieve long‐term remission and those who respond more favorably to immunomodulators.

## 5 Conclusion

This study suggests that rituximab may contribute to longer intervals between airway interventions in GPA‐associated laryngotracheal stenosis and, in some cases, complete remission of proximal airway disease progression, supporting the role of systemic immunosuppression in disease stabilization. While further research is needed, these findings emphasize the importance of comprehensive disease control and multidisciplinary coordination in managing this challenging, life‐threatening manifestation of GPA.

## Conflicts of Interest

The authors declare no conflicts of interest.

## Data Availability

The data that support the findings of this study are available from the corresponding author upon reasonable request.
